# Boosting the Mechanical Strength and Photocatalytic Activity of 3D-Printed Titania Aerogels by Atomic Layer Deposition and Heat Treatment

**DOI:** 10.1021/acsmaterialsau.5c00162

**Published:** 2025-11-05

**Authors:** Malte M. Schmidt, Tjark L. R. Gröne, Robert Zierold, Diego Ribas Gomes, Sandra König, Michael Fröba, Kaline P. Furlan, Dorota Koziej

**Affiliations:** † Center for Hybrid Nanostructures (CHyN), Institute for Nanostructure and Solid State Physics, 14915 University of Hamburg, Luruper Chaussee 149, 22761 Hamburg, Germany; ‡ Institute of Advanced Ceramics, Hamburg University of Technology (TUHH), Denickestr. 15(K), 21073 Hamburg, Germany; § The Hamburg Centre for Ultrafast Imaging, Luruper Chaussee 149, 22761 Hamburg, Germany; ∥ Institute of Inorganic and Applied Chemistry, University of Hamburg, Martin-Luther-King-Platz 6, 20146 Hamburg, Germany

**Keywords:** 3D printing, titania, aerogels, atomic layer deposition, mechanical properties, photocatalysis, water splitting

## Abstract

Titania aerogels are highly porous materials optimal for photocatalysis due to their high surface area. Further spatial structuring by 3D printing improves gas diffusion in the aerogel, leading to a higher photocatalytic activity. However, the aerogel’s mechanical properties are reduced in comparison to non-3D printed aerogels. We hereby present an approach based on atomic layer deposition (ALD) of subnanometer-thin TiO_2_ layers to compensate for that detrimental effect. The ALD-deposited TiO_2_ consists of amorphous and anatase phase, with the anatase phase likely crystallizing on the aerogel’s crystallites. Nanoindentation measurements confirm that the TiO_2_ ALD-coatings improve the aerogel’s mechanical properties. Additionally, it enhances the photocatalytic properties of the TiO_2_ aerogel, which we attribute to the increased interface area and improved interconnection of the nanoparticle network. By further thermal postprocessing, it is possible to fully crystallize the ALD-deposited TiO_2_, which shows a complementary effect on photocatalytic performance, improving hydrogen evolution rate by more than 1 order of magnitude from 6.35 to 125 μmol g^–1^ h^–1^. The combination of 3D structuring of aerogels with ALD coatings demonstrated in this work could be extended in the future to a wide range of materials where the interplay between mechanical and catalytic properties is vital.

## Graphical abstract

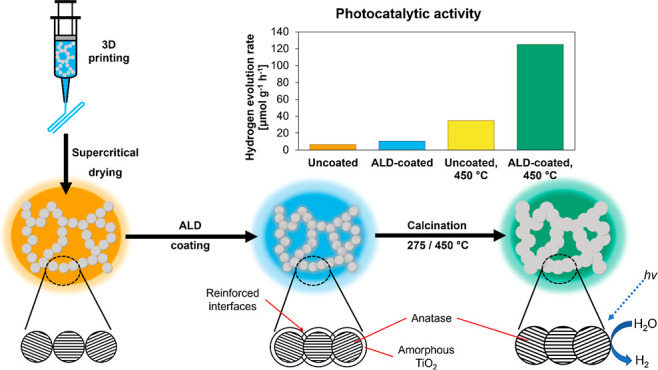

## 1 Introduction

Aerogels are nanoporous, low-density materials with unique properties such as high surface area, high porosity, and low thermal conductivity.^1^ These characteristics, derived from their nanostructured framework, make aerogels promising candidates for applications such as thermal and acoustic insulation,^2^ gas filtration,^3^ catalysis,^2^ and photocatalysis.^4^ Although aerogels appear solid on the macroscopic scale, they predominantly consist of nanopores that account for most of their volume.^3^ Their pore walls are made from nanoscale building blocks, synthesized either via a sol–gel process or by assembling preformed nanoparticles.^5^ The hierarchical architecture of aerogels ensures that the size-dependent properties of their nanoscopic building blocks are preserved in the macroscopic form.^6^

Titanium dioxide (TiO_2_) is a widely researched material known for its effectiveness as a photocatalyst.^7^ A prominent model reaction studied in photocatalysis is water splitting,^8^ first reported using TiO_2_ by Fujishima and Honda in 1972.^9^ TiO_2_ functions as an n-type semiconductor with a wide bandgap,^10^ exhibiting three common crystalline polymorphs—rutile, anatase, and brookite—in which titanium atoms are octahedrally coordinated by oxygen atoms.^11^ While rutile is the thermodynamically stable phase in bulk form, anatase dominates in nanoparticle-based systems, i.e., aerogels, due to its lower surface energy.^12^ Anatase also exhibits superior photocatalytic performance due to its indirect bandgap (3.2 eV), which reduces charge carrier recombination and, hence, enables longer electron–hole lifetimes.^13^ Given its high surface area and favorable band structure, hierarchically structured TiO_2_ aerogels are promising candidates for photocatalysis.

To control the structure of aerogels across different length scales, extrusion-based 3D-printing techniques, such as direct ink writing (DIW), offer precise structural control.^14^ One example of this additive manufacturing approach is the 3D-printed TiO_2_ nanoparticle aerogel^15^ used in this study. The DIW method allows structuring on the microscale, bridging the nanoscale features of aerogels and their macroscale form, thereby enabling the fabrication of hierarchical architectures.^16^ Such hierarchical structuring not only preserves the high surface area and porosity of aerogels but also improves performance by enhancing mass transport, light harvesting, and accessibility to catalytic sites, while reducing diffusion limitations and facilitating gas release during photocatalysis.^17^ Thereby, spatial structuring of TiO_2_ across different length scales has been demonstrated to enhance its photocatalytic performance.^18^ For instance, 3D-printed TiO_2_ aerogels have shown 5-fold higher hydrogen evolution rates compared to nanoparticle powders.^17^ In that study, a woodpile structure enhanced gas permeability compared to a monolithic aerogel, without compromising light-harvesting efficiency.^17^

Despite their functional advantages, aerogels face limitations regarding their mechanical properties. Although they can withstand compressive stress corresponding to many times their own weight due to their branched microstructure, aerogels are still considered highly brittle, which limits their practical applications.^2,19^ Conventional approaches to improve mechanical stability, such as sintering, lead to partial densification but at the expense of reducing porosity and surface area, which in turn can negatively impact the photocatalytic performance.^3^

A promising alternative to address these challenges is atomic layer deposition (ALD), a thin-film deposition technique known for producing conformal coatings onto complex structures without shadowing effects.^20^ Therefore, ALD is ideal for coating porous substrates with high surface area, such as aerogels, and the improved precursor diffusion due to 3D structuring makes the method particularly applicable to our 3D-printed TiO_2_ aerogels. Al_2_O_3_ ALD coatings have been previously shown to improve the mechanical stability of nanoporous materials, including aerogels.^21,22^ In this study, we investigate the effect of TiO_2_ ALD coatings on both the mechanical properties and photocatalytic performance of 3D-printed TiO_2_ aerogels. These ALD coatings, which are typically polycrystalline or amorphous depending on the ALD cycle conditions,^23^ can change the phase composition and surface properties, which can result in different charge carrier transport.^24^

The fabrication and functionalization steps for the 3D-printed TiO_2_ aerogels are outlined in Figure 1. After gelation, TiO_2_ solvogels were either gel-cast as monolithic gels or 3D-printed via DIW in a woodpile structure with shifted layers, according to a previously reported procedure.^15^ After supercritical CO_2_-drying to remove the solvent, the aerogels were subjected to ALD-coating for structural reinforcement and calcination at 275 or 450 °C to crystallize amorphous TiO_2_.

**Figure 1:**
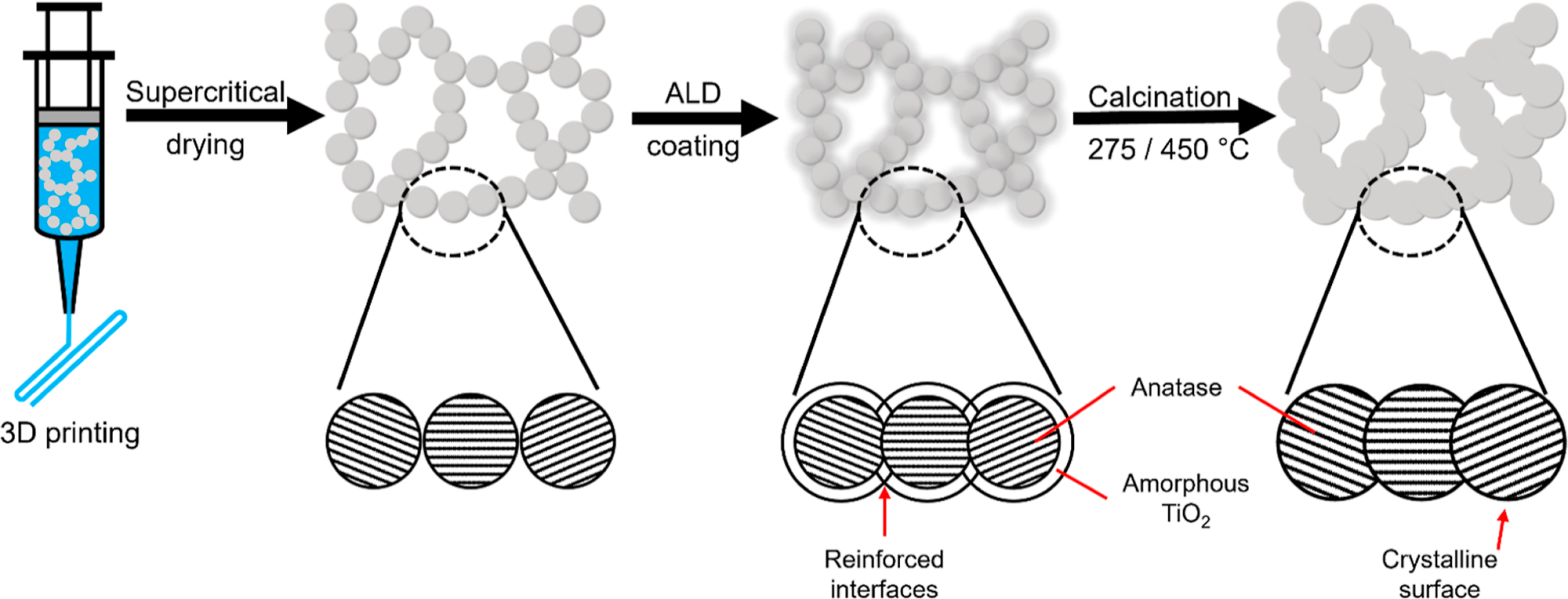
Fabrication scheme for mechanically stable and highly photoactive titania aerogels: This includes the 3D printing of nanoparticle-based gelled ink, supercritical drying, atomic layer deposition (ALD) coating of the resulting aerogel, and calcination at various temperatures. The possible transformations occurring at the grain boundaries of the particles during each of these fabrication steps are illustrated in the respective insets below.

## 2 Results and Discussion

To evaluate the structural characteristics of the TiO_2_ aerogels, scanning electron as well as transmission electron microscopy (SEM, TEM) and N_2_ physisorption measurements were performed on uncoated and ALD-coated aerogels before and after heat-treatment at 450 °C.

SEM images (Figures S1b and S2) reveal a highly porous, homogeneous structure with pore sizes of approximately 20 nm. No apparent differences are visible in the SEM and TEM (Figure S2a–d) between the uncoated and the ALD-coated aerogels, indicating that the ALD-deposited TiO_2_ layer is extremely thin and conforms uniformly to the porous framework without altering its macroscopic morphology. Due to a lack of material contrast, detailed thickness determination is not possible. N_2_ physisorption measurements confirm the minimal impact of the ALD coating on the overall aerogel structure. Specifically, the uncoated and ALD-coated aerogels retain similar pore sizes of approximately 22 nm and surface areas larger than 600 m^2^/g as shown in Figure 2a,b and Table S1. We attribute the increase in surface area after ALD coating to variability between initial uncoated samples. The TiO_2_ aerogels display a type IVa isotherm with an H1 hysteresis loop, indicating a mesoporous adsorbent with a narrow range of uniform mesopores.^25^ The layer deposited inside the aerogel might be thinner than a layer deposited on a planar reference sample because of the limited diffusion and the intrinsic inhibition behavior of the titania ALD process.^26–28^ The calcined and ALD-coated and calcined aerogels exhibit significantly larger nanoparticles, a smaller average pore size of around 16 and 18 nm and a surface area of 400 m^2^/g and 200 m^2^/g, respectively as shown in Figures S2e,f and S3 and Table S1.

**Figure 2:**
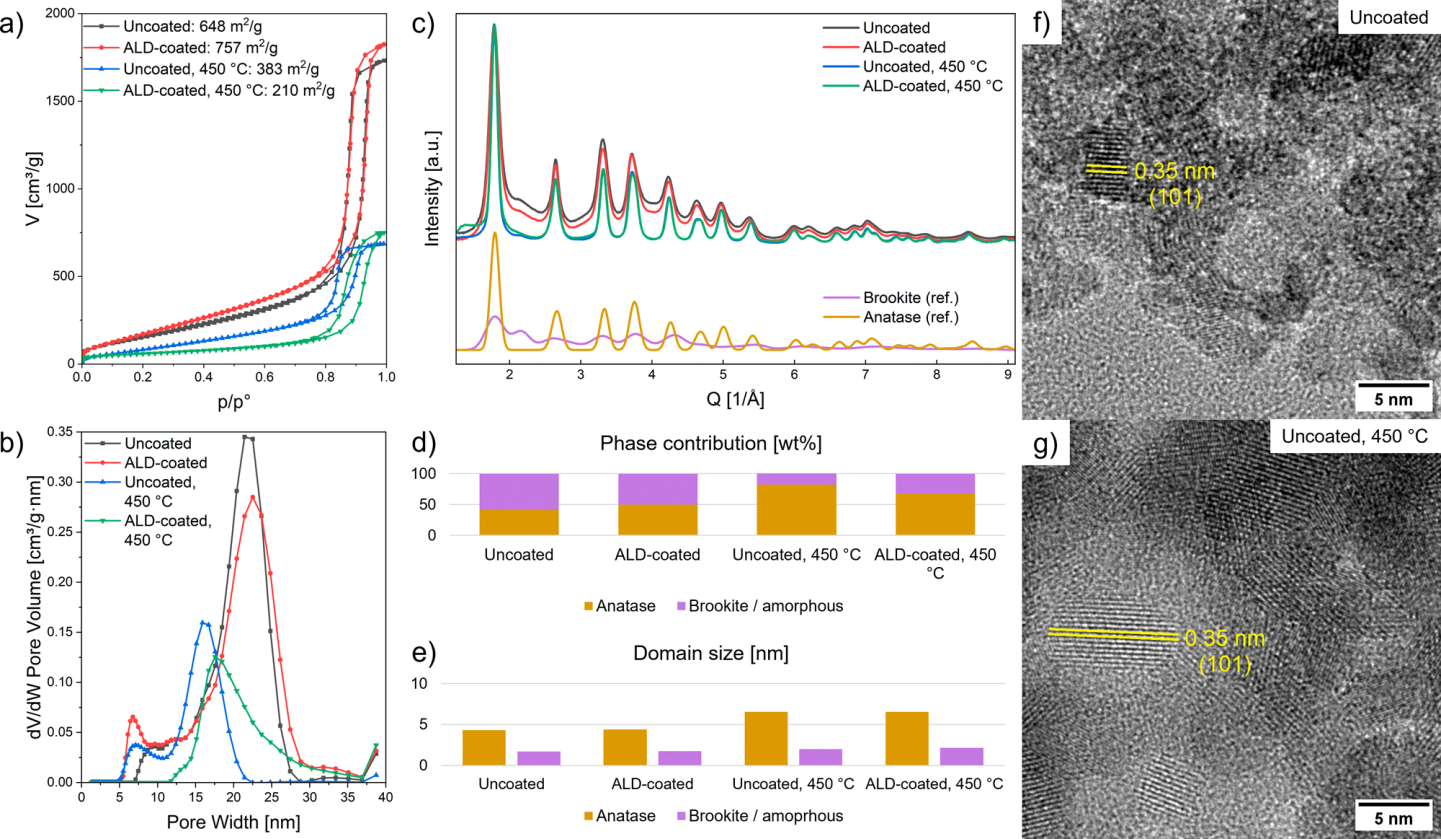
(a) Nitrogen physisorption isotherms for an uncoated and an ALD-coated aerogel before and after calcination at 450 °C. (b) Corresponding pore size distributions determined by DFT analysis of the desorption branches. Table S1 lists the samples’ mean pore size and specific surface area. (c) PXRD patterns of the same aerogels and reference patterns calculated for brookite (2 nm) and anatase (5 nm). The experimental PXRD patterns were fitted with a mixture of anatase and brookite using Rietveld refinement (Figure S5). The phase weight contributions (d) and domain sizes (e) of anatase and brookite were determined from these fits. HR-TEM images of an uncoated aerogel before (f) and after (g) calcination at 450 °C.

High resolution (HR)-TEM images reveal small crystallites of ∼4 nm embedded within an amorphous matrix prior to calcination as shown in Figure 2f. There are no significant differences between the uncoated and ALD-coated aerogel, with crystallite sizes of mostly about 4 nm but also a small number of bigger crystallites as shown in Figure S4. The absence of apparent features from the ALD layer confirms its ultrathin nature, likely an amorphous thin film filling up nanoparticle gaps rather than forming an overcoating layer with distinct crystalline grains (Figure S4b,d). After calcination, the crystallite size increases to ∼6 nm, consistent with sintering and phase transformation as shown in Figure 2g.

Powder X-ray diffraction (PXRD) measurements provide quantitative insight into phase evolution (Figure 2c–e). The main reflexes correspond to anatase, while shoulders at 2.4 Å^–1^, 3.1 Å^–1^, and 4.0 Å^–1^ could correspond to small brookite crystallites or amorphous TiO_2_.^29^ There are limitations to PXRD fitting due to the extensive peak broadening at these small domain sizes. While the two-phase fit with Rietveld refinement suggests a significant contribution from brookite, this result may partially reflect the presence of amorphous TiO_2_, given the similar diffraction patterns between small brookite crystallites and amorphous phases.^29^ The relative contributions of amorphous TiO_2_ and ultrasmall brookite nanoparticles remain difficult to resolve using PXRD alone.

The two-phase fit, considering anatase and brookite, of the PXRD pattern with Rietveld refinement results in a mixed phase of anatase of 41.2 wt % with small domain sizes of ∼4.3 nm and brookite 58.8 wt % with tiny domain sizes ∼1.7 nm for the uncoated aerogel. In general, the mixed-phase compositions, such as anatase combined with brookite, can exhibit synergistic effects leading to enhanced photocatalytic performance compared to pure phases.^30,31^ After ALD-coating, the anatase content increases to 49.9 wt %, indicating some degree of epitaxial growth of crystalline anatase on pre-existing domains, likely driven by the deposition process. The presence of anatase in ALD-deposited TiO_2_ agrees with a previous report, where similar deposition conditions were used.^32^ After calcination, the anatase fraction grows considerably to 82.3 wt %, accompanied by a domain growth to ∼6.5 nm for both coated and uncoated samples, which is consistent with the HR-TEM analysis. These findings suggest that ALD improves crystallinity while preserving amorphous regions that can be converted to anatase during calcination, enhancing photocatalytic activity.

We investigate the mechanical properties of the aerogels utilizing nanoindentation measurements to determine hardness and elastic modulus as shown in Figure 3. Compared to gel-cast monolithic aerogels, the 3D-printed aerogels show a reduction in hardness of about 40% and also in elastic modulus of about 35%. This is attributed to shear-induced disruption of the aerogel nanostructure during extrusion-based printing,^33^ which likely introduces microcracks and reduces particle connectivity, compromising mechanical integrity.

**Figure 3:**
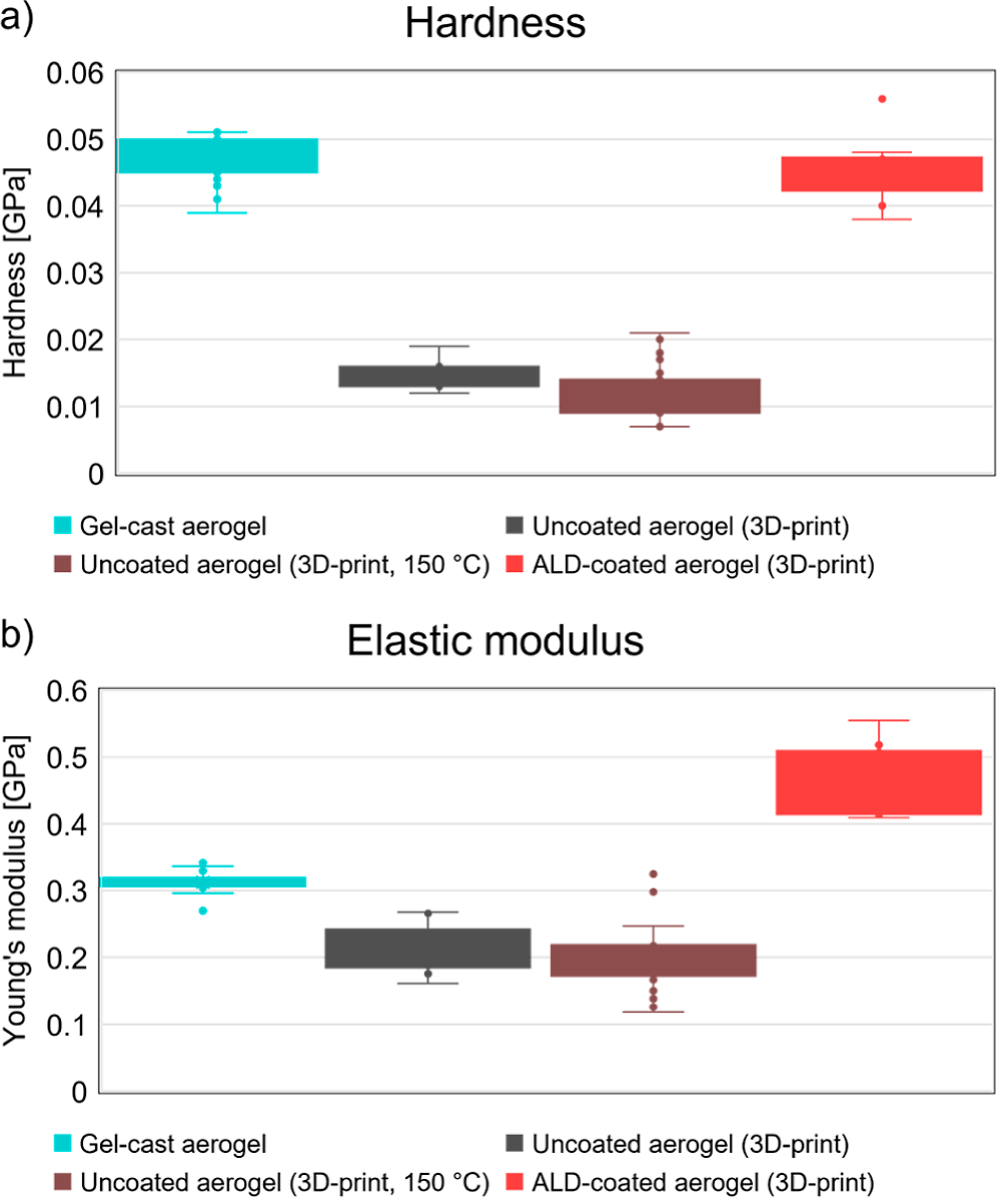
Hardness (a) and Young’s modulus (b) of a gel cast, monolithic aerogel (aquamarine), an uncoated, 3D-printed aerogel (dark gray), an uncoated, 3D-printed aerogel heat treated under similar conditions as ALD-coating (brown), and an ALD-coated, 3D-printed aerogel (red). The mechanical properties were determined by nanoindentation measurements with a spherical tip in continuous stiffness mode. The corresponding load-displacement curves are shown in Figure S6.

However, the ALD-coated aerogels exhibit a significant recovery in mechanical properties, with modulus values exceeding those of the monolithic gel-cast aerogels. This enhancement can be attributed to the conformal nanoscale TiO_2_ coating. On one side, it is expected to fill microcracks and nanoparticle gaps, effectively reducing potential stress concentrators and reinforcing particle interfaces. On another side, it provides additional stiffness due to the high modulus of the TiO_2_ coating, as observed in prior studies of ALD-coatings improving mechanical stability in nanoporous materials.^21,22^ The narrower interquartile range (IQR) for ALD-coated samples indicates greater uniformity in mechanical reinforcement, supporting the hypothesis that ALD improves network connectivity across the aerogel structure and enhances load distribution. Note, since the ALD process involves prolonged heating at 150 °C in a vacuum, it was necessary to rule out the possibility that the observed increased mechanical strength resulted from thermal densification rather than the coating itself. To isolate the potential contribution from the heating at 150 °C, an uncoated aerogel was exposed to the same thermal conditions as the ALD process. As the hardness and elastic modulus are not altered by the sole ALD-mimicking heat treatment at 150 °C (Figure 3, brown boxes), it confirms that the improved mechanical properties observed in ALD-coated samples arise from the coating’s structural reinforcement and interface strengthening.

Both uncoated and ALD-coated aerogels exhibit changes in hardness and modulus upon calcination at 275 and 450 °C as shown in Figure S7. For uncoated aerogels, both properties increase with calcination temperature, consistent with densification due to sintering. This structural consolidation enhances mechanical strength but leads to a ∼41% reduction in surface area, as indicated by BET measurements (Table S1). In contrast, ALD-coated aerogels exhibit distinct mechanical behavior: At 275 °C, their hardness surpasses that of uncoated aerogels calcined under the same conditions, indicating that the TiO_2_ coating reinforces the aerogel network. However, at 450 °C, hardness decreases significantly, suggesting thermally induced degradation or stress relaxation within the coated network. Notably, the elastic modulus of ALD-coated aerogels remains relatively stable across both temperatures, implying that the TiO_2_ layer may act as a structural stabilizer while restricting densification by impeding particle rearrangement. Despite this restriction, ALD-coated aerogels retain a higher porosity (600 m^2^/g) while achieving mechanical properties comparable to those of densified, uncoated aerogels. This result highlights the dual advantage of ALD-coatings, which enhance mechanical integrity of the aerogel network while preserving high surface area, a critical attribute for catalytic applications.

Furthermore, we demonstrate the material’s photocatalytic activity for water splitting, using methanol as a sacrificial agent to scavenge holes. The photocatalytic measurements were performed in a gas-phase reactor setup described previously.^17^ Water and methanol vapor are introduced into the photoreactor using helium as a carrier gas, where a 375 nm UV LED, matching the bandgap of the material (Figure S8a), illuminates the 3D-printed TiO_2_ aerogel with a woodpile structure (Figure 4a,b). The resulting hydrogen gas is quantified in a gas chromatograph and plotted against time for a total test duration of 24 h (Figure 4d).

**Figure 4:**
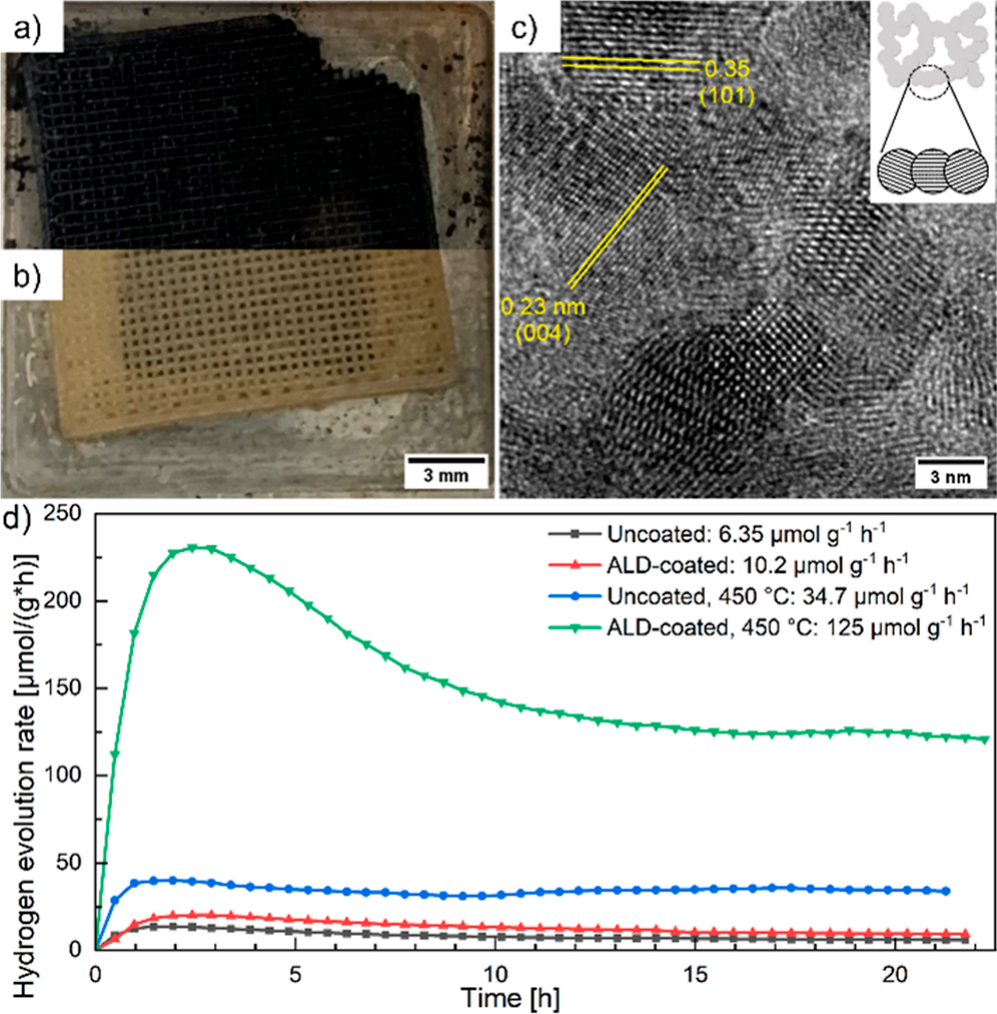
Photograph of a discolored 3D-printed TiO_2_ aerogel after UV illumination in a helium gas stream (a), and the same aerogel after re-exposure to air (b). HR-TEM image (c) of the same aerogel, which was ALD-coated and calcined at 450 °C, and showed the best photocatalytic performance. The inset shows the schematic nanostructure of this aerogel. (d) Hydrogen evolution rate as a function of time for an uncoated, an ALD-coated, uncoated calcined, and ALD-coated calcined TiO_2_ aerogel.

The hydrogen evolution rate goes through a maximum at the beginning of each measurement before reaching a steady-state value after about 10 h. This initial peak can be explained by the surface coverage of initially free surface sites, primarily by intermediates from the degradation of methanol, which leads to a reduction of surface sites and photocatalytic activity until an equilibrium is reached.^34^

Overall, the TiO_2_ aerogels exhibit high photocatalytic activity, outperforming the benchmark material TiO_2_ P25 under the same measurement conditions (Figure S8b). This high performance is attributed to the large surface area in the aerogel, along with to the optimized mass transport and light utilization facilitated by the 3D structuring.^17^ The phase composition containing both anatase and brookite may also contribute significantly to the high photocatalytic performance. This mixed-phase TiO_2_ system has been shown to outperform pure anatase, pure brookite, and even TiO_2_ P25 due to favorable band alignment between the two polymorphs,^30,31^ which promotes charge separation and reduces electron–hole recombination.

Furthermore, we observed a color change of the TiO_2_ aerogels during the photocatalysis measurements. During illumination with UV irradiation under inert helium gas, the color of the TiO_2_ aerogels turns blueish-black. This discoloration is completely reversible: after being exposed to air again, the aerogels’ color changes back within seconds (Figure 4a,b). Black titania is a well-known modification of TiO_2_ that exhibits a black color due to the excess of oxygen vacancies and lattice disorder,^6,35^ although the color change usually occurs under more harsh conditions and is not reversible.^36^ Blue titania has been previously reported after UV irradiation in an inert atmosphere and is explained by free conduction band electrons reducing some Ti^4+^ ions to Ti^3+^.^37^ In the presence of oxygen, the Ti^3+^ ions are quickly oxidized again and the blue color is quenched.^38^ Similarly, we assign the reversible formation of black titania to the excitation of electron–hole pairs and a reversible reduction of Ti^4+^ ions by conduction band electrons.

We observe a significant increase in hydrogen production rate for both uncoated and ALD-coated aerogels after calcination. This can be explained by the increased crystallinity as visible in the PXRD data (Figure 2c–e), resulting in more catalytically active surface sites. Therefore, a high crystallinity is beneficial for photocatalytic performance.^8^ Moreover, the improvement upon calcination could also receive a contribution from the removal of organic residues from synthesis and fabrication of the aerogel that would otherwise block surface sites. It was previously shown that organics are not completely removed during UV cleaning.^17^ ALD-coating also increased the hydrogen evolution rate, both before calcination on a low level from 6.35 ± 0.38 μmol g^–1^ h^–1^ for the uncoated aerogel to 10.2 ± 0.85 μmol g^–1^ h^–1^ for the ALD-coated aerogel, and after calcination from 34.7 ± 0.5 μmol g^–1^ h^–1^ for the uncoated aerogel to 125 ± 2.3 μmol g^–1^ h^–1^ for the ALD-coated aerogel, averaged over the last 20 measurement points. Remarkably, this enhancement is comparable to the previously reported effect of loading 3D-printed TiO_2_ aerogels with 0.9 wt % Au nanoparticles (142 ± 10 μmol g^–1^ h^–1^),^17^ demonstrating the combined effectiveness of ALD-coating and calcination for boosting photocatalytic activity without the need for noble metal cocatalysts.

A possible explanation for the improved photocatalytic performance upon ALD-coating is an improved interconnectivity between the nanoparticles. The deposited TiO_2_ layer may passivate surface defects on the TiO_2_ nanoparticles, reducing the probability of charge carrier recombination and trapping.^39^ However, the ALD-deposited TiO_2_ could also introduce new surface defects, counteracting the passivation effect. Moreover, the TiO_2_ can enhance carrier mobility by lowering the energy barrier for interparticle transport.^39^ The gaps between the nanoparticles are filled, therefore offering more pathways for the charge carriers to reach catalytic sites. This mechanism has been previously proposed for an ALD-coated TiO_2_ nanoparticle photoelectrode in a dye-sensitized solar cell.^40^ Furthermore, the theoretical work on the metal–insulator transition in semiconductor nanocrystal networks generally supports the idea that the interface consists of small facets where two nanocrystals contact, showing that conductance scales with the contact area between nanocrystals. Thus, increasing the contact interface area by ALD-coating possibly reduces the localization of charge carriers and improves the photocatalytic activity of the TiO_2_ aerogel.^41^ Additionally, other studies indicate that crystallinity and interfacial connectivity are crucial for the conductivity of semiconductor nanocrystal assemblies, with epitaxial connections resulting in significantly higher conductivity and band-like transport compared to ligand-bridged nanocrystals, which exhibit hopping transport.^42,43^ Even though the ligand-free TiO_2_ nanoparticles are not epitaxially connected in the gel, calcination crystallizes the amorphous parts of the TiO_2_ nanoparticles and the deposited TiO_2_ layer. For an ALD-coated aerogel, this leads to larger crystalline interfaces between the nanoparticles (Figure 4c), thus most likely further reducing charge carrier recombination and trapping, and explaining the particularly high photocatalytic activity.

## 3 Conclusions

In this study, we developed a 3D-printed TiO_2_ nanoparticle aerogel with a hierarchical woodpile structure and demonstrated the use of ALD to enhance its mechanical strength and photocatalytic performance. ALD enables the coating of internal surfaces with a sub-nm TiO_2_ layer, while preserving the high surface area and mesoporous structure of the aerogel.

The initial TiO_2_ nanoparticle aerogels comprised small anatase and brookite domains embedded within amorphous material. The ALD-deposited TiO_2_ initially forms as a mixture of amorphous material and anatase, with the anatase fraction likely nucleating on pre-existing anatase crystallites. Upon calcination, the amorphous regions are converted into anatase, increasing overall crystallinity.

Nanoindentation measurements confirmed that the 3D-printing process reduced the aerogel’s hardness and modulus due to shear-induced disruption of the nanoparticle network. However, ALD-coatings restored mechanical strength, achieving values comparable to calcined aerogels while preserving porosity and surface area. This improvement was attributed to the filling of microcracks and interface reinforcement by the TiO_2_ coating, offering mechanical stability without compromising the high surface area needed for catalytic activity.

Our results show that ALD-coating significantly enhances the photocatalytic activity for hydrogen production in a gas-phase reactor, probably caused by an improved interconnectivity and larger interface areas between the nanoparticles. Calcination further increases the photocatalytic activity by removing organic residues on the particle surface and increasing crystallinity. ALD coating and calcination complement each other in enhancing photocatalytic performance by creating larger crystalline interfaces that may improve charge carrier transport. The combined effects increase the hydrogen evolution rate by more than 1 order of magnitude compared to the uncoated TiO_2_ aerogel. Our method illustrates the great potential of 3D structuring of aerogels in combination with ALD coatings. In the future, this approach could be applied to a diverse array of structured materials where the interaction between mechanical and catalytic properties plays a crucial role.

## 4 Experimental Section

### 4.1 TiO_2_ Synthesis

The synthesis of TiO_2_ nanoparticles was adapted from a previously published protocol.^44^ In short, 5 mL of anhydrous ethanol was cooled in a water bath, while 1 mL of TiCl_4_ was slowly added, resulting in a transparent yellow solution and the release of HCl gas. Then, the TiCl_4_ solution was added to 20 mL of anhydrous benzyl alcohol that had been preheated. The mixture was stirred at 80 °C for 24 h in an open flask. The synthesis resulted in an opaque suspension, which was cooled to stop the reaction. The mixture was separated through centrifugation at 1160*g* for 5 min, with diethyl ether used to precipitate the nanoparticles. After three cycles of centrifugation, the product was air-dried and ground into a fine powder. This TiO_2_ powder was weighed and redispersed in Milli-Q water at a concentration of 242 mg/mL for storage in a fridge.

### 4.2 Gelation

Acetonitrile (ACN) was added to the TiO_2_ nanoparticle dispersion in a 2:1 volume ratio of water to ACN within a sealed 5 mL syringe. The mixture was then homogenized using a planetary centrifugal mixer (THINKY ARE-250) for two 2 min cycles at 1500 rpm with one defoaming step in between. Following this, the syringe was inverted and centrifuged at 210*g* for 1 min. After removing the syringe cap, the dispersion was gelled in an oven at 60 °C for 17.5 min, resulting in a solid, gel-like consistency. This solvogel could be stored at room temperature for up to 2 days for 3D printing.

For producing a monolithic gel, the syringe was used as a mold for gel casting. The upper part of the syringe was cut off and placed upside down on the remaining syringe. Then the syringe was placed in the oven at 60 °C for 17.5 min. After gelation, the plunger was slowly pushed out to detach the cylindrical solvogel monolith, which was then immersed in a 2:1 mixture of water and ACN for the solvent exchange process and supercritical drying described in 4.4. Supercritical drying.

### 4.3 3D Printing

The 3D printing process is described in detail in a previously published protocol.^15^ In short, the TiO_2_ solvogel was extruded onto a glass substrate by direct ink writing (DIW). The substrates were coated with two polymer layers: one polyvinylpyrrolidone (PVP) layer followed by one poly­(methyl methacrylate) layer. The TiO_2_ gel was extruded from a syringe through a conical nozzle from Vieweg GmbH with a diameter of 250 μm. Using an Engine HR 3D printer (Hyrel 3D, USA), the gel was deposited onto the substrate in a printing container in a woodpile structure. This container was filled with a liquid bath of heptane enriched with ammonia. Every 5 min during the printing process, the liquid bath was refreshed with newly ammonia-enriched heptane. After printing the gel, the printing container was filled with ammonia-enriched heptane, covered, and left undisturbed for 90 min, allowing the structure to harden. The gel was then detached from the substrate by immersing it in a 2:1 mixture of water and ACN, effectively dissolving the PVP coating. After the detachment, the structure underwent further curing in ammonia-enriched heptane for 24 h.

### 4.4 Supercritical Drying

Following gelation and 3D printing of the gel, a solvent exchange was carried out, involving a stepwise transition to ethanol. To achieve this, the samples were immersed in a sequence of solvent mixtures with the following volume ratios: H_2_O/ACN = 2:1, 1.4:1, 40:60, 30:70, 20:80, 10:90, 0:100, followed by ACN/ethanol = 50:50, 0:100, with at least 7 h between each solvent exchange. Following ethanol immersion, the ethanol was replaced with liquid CO_2_ in a Quorum E2100 supercritical dryer. The transition to pure CO_2_ was performed by draining the CO_2_/ethanol mixture multiple times and then refilling the boat with pure CO_2_. The dryer temperature was increased to 40 °C, resulting in a pressure of 90 bar, to convert the liquid CO_2_ into a supercritical state before gradually releasing the pressure at a rate of 0.5–1.0 bar/min to obtain dry aerogels.

### 4.5 ALD-Coating

Some of the TiO_2_ aerogels were coated with a thin TiO_2_ layer using atomic layer deposition (ALD) in a homemade ALD reactor. Initially, the aerogels were outgassed at 150 °C for 2 h. Then, 75 ALD cycles were executed at 150 °C with a nitrogen flow rate of 25 ccm. Each ALD cycle involved pulsing titanium tetraisopropoxide heated at 85 °C for 2 s and pulsing water at room temperature for 0.5 s. The exposure time for both precursors was 60 s, followed by a pumping period of 90 s. The growth process was controlled by adding a planar silicon wafer next to the aerogel. After running the process, the thickness was determined by spectroscopic ellipsometry to be about 2 nm.

### 4.6 Heat Treatments

Some of the 3D-printed titania nanoparticle aerogels were calcined in a tube furnace by Carbolite. Calcination was performed at 450 or 275 °C for 30 min with a heating rate of 2 °C/min. To replicate the conditions of the ALD process, the titania aerogels were heated in a vacuum oven by Memmert at 150 °C for 18 h.

### 4.7 Microscopic and Spectroscopic Characterization

The TiO_2_ aerogels were ground to a powder and deposited on copper grids for electron microscopy imaging. Scanning electron microscopy images were taken with a Regulus 8220 (Hitachi High Technologies Corp., Japan). Transmission electron microscopy images were taken with a JEM 1011 (JEOL Ltd., Japan). High-resolution TEM images were taken with a JEM-2200FS (JEOL Ltd., Japan). Optical microscopy images were taken with a VK-X3000 3D laser scanning microscope (Keyence, Japan). UV/vis absorbance spectra of aerogel plates were measured using a Cary 5000 spectrophotometer (Agilent, USA) with an Agilent DRA-2500 integrating sphere.

### 4.8 N_2_ Physisorption

Nitrogen physisorption measurements of about 50 mg 3D-printed TiO_2_ aerogels were performed at 77 K using a 3Flex Analyzer (Micromeritics Instrument Corp., USA). Prior to the measurements, the samples were activated at 100 °C for 16 h under reduced pressure using a Smart VacPrep Degasser (Micromeritics Instrument Corp., USA). For data reduction, the software MicroActive, Version 6.00, was used. The total pore volume was calculated at 0.98 *p*/*p* ^0^. To determine the specific surface area the Brunauer–Emmett–Teller (BET) method was used at a *p*/*p* ^0^ range from 0.05 to 0.30. Calculations of pore size distribution were done by using a density functional theory (DFT) model for oxide surface chemistry with cylindrical pore geometry.

### 4.9 Powder X-ray Diffraction

The powder X-ray diffraction ex situ data of the aerogels were taken at Beamline P21.1 at PETRA III DESY in Hamburg, Germany by a Perkin detector model XRD1621 (PerkinElmer Inc., USA) with a total of 4096 × 4096 pixels and a size of 200 × 200 μm. The 2D data were azimuthally integrated with the Python software PyFAI.^45^

The uncoated, ALD-coated, and uncoated 450 °C aerogels were measured with an X-ray beam energy of 101.387 keV at an SDD of 0.980 m and a beam tilted by 0.118 deg and the beam center at *x* = 1009.501, *y* = 1005.709 pixels. The ALD-coated 450 °C aerogels were measured with an X-ray beam energy of 101.38 keV at an SDD of 1.487 m and a beam tilted by 0.134° and the beam center at *x* = 1035.505, *y* = 1027.937 pixels. All samples were ground to a powder, enclosed in 1 mm Kapton capillaries and calibrated by a crystalline powder sample of LaB_6_. An empty Kapton capillary was subtracted from each data set as a background.

Rietveld refinement of the X-ray diffraction data was performed with DiffracSuite Topas V6 software (Bruker AXS GmbH, Karlsruhe, Germany).^46^ The instrumental resolution parameters for peak broadening and TCHZ profile were determined from a fit to the experimental standard of LaB_6_ by the Crystallography Open Database (COD) reference 2104747 (*Pm̅*3*m*, 221). Models for a nanoparticle anatase phase COD 1526931 (*I*4_1_/*amd*:2, 141) and brookite phase COD 9004140 (*Pbca*, 61) were fitted to the aerogel data. With the refined parameters, we determined the phase weight percentage, lattice parameters, domain size, micro strain, and isotropic thermal displacement parameter *B*_eq_.

### 4.10 Nanoindentation

Nanoindentation measurements were performed using an Agilent Nano Indenter G200 equipped with a spherical tip (10 μm diameter). The continuous stiffness measurement (CSM) mode was utilized, operating with a displacement amplitude of 2 nm and a frequency of 45 Hz. The strain rate during indentation was maintained at 0.05 s^–1^. To minimize the influence of the indentation size effect, a prior depth-dependent analysis was conducted. Based on this analysis, an indentation depth of 1.5 μm was selected for testing. The mechanical properties, including hardness and elastic modulus, were calculated by averaging the data collected within the depth range of 1.2 to 1.35 μm (corresponding to 70–90% of the total indentation depth). For woodpile-structured samples, indents were specifically positioned at the center of the struts to minimize the influence of lateral forces and edge effects on the measurements. All measurements were repeated at least five times at different positions on each sample to ensure statistical reliability. The results are presented as boxplots, displaying the interquartile range (IQR), median values, and potential outliers, which are identified as data points lying outside 1.5 times the IQR.

### 4.11 Photocatalytic Water Splitting

We measured photocatalytic activity using a custom-built gas-phase photoreactor. This setup was previously described.^17^ It incorporates mass flow controllers (Bronkhorst, Netherlands) to regulate gas flow up to 20 mL/min. The carrier gas first flows through a saturation chamber filled with solvents to enrich it with their vapors before the gas enters the photoreactor. Aerogel samples are positioned on a sample tray with a square hole and sealed with grease below the sample edges, allowing optimal gas flow through the sample and illumination through a reactor window. The gas exiting the reactor is analyzed using a gas chromatograph (8860 GC System, Agilent Technologies, USA) equipped with a PoraPLOT U column and an HP-Plot 6A column for separation of the gas components and with a helium ionization detector and a thermal conductivity detector for detection. The gas mixture is injected into the gas separation system every 29 min, giving the time resolution of the measurement.

Before each measurement, samples underwent a UV cleaning step. This involved synthetic air flushing for 24 h at 5 mL/min under illumination from a mercury/xenon arc lamp (66984-200HX-R1, Newport, USA) with a water filter. Following this, the saturation chamber was filled with 30 mL of a mixture of water and methanol (1:1 v/v) as the reactant source. The setup was flushed with helium at 20 mL/min to remove residual air. Then, the flow rate was reduced to 5 mL/min and the illumination from a 375 nm UV LED (M375L4, Thorlabs) was turned on. Photocatalytic measurements ran for 24 h.

## Supplementary Material



## Notes

Light microscopy, SEM, and TEM images of uncoated and ALD-coated TiO_2_ aerogels; surface area and pore size information from N_2_ physisorption measurements; HR-TEM and PXRD patterns before and after calcination; load-displacement curves and mechanical property data from nanoindentation measurements; UV/vis absorbance spectra and photocatalytic hydrogen evolution measurements with TiO_2_ P25 powder as reference (PDF)

## #

Karlsruhe Institute of Technology (KIT)—Campus Süd, Haid-and-Neu Straβe 7, 76131 Karlsruhe, Germany

## Abbreviations

ACN: acetonitrile
ALD: atomic layer deposition
BET: Brunauer–Emmett–Teller
CSM: continuous stiffness measurement
DFT: density functional theory
DIW: direct ink writing
HR: high resolution
IQR: interquartile range
PVP: polyvinylpyrrolidone
PXRD: powder X-ray diffraction
SEM: scanning electron microscopy
TEM: transmission electron microscopy
