# Supplementary material for: Boosting the Mechanical Strength and Photocatalytic Activity of 3D-Printed Titania Aerogels by Atomic Layer Deposition and Heat Treatment
Source: ACS Mater Au. 2025 Nov 5;6(1):213–21. doi: 10.1021/acsmaterialsau.5c00162 (PMC12810030; doi:10.1021/acsmaterialsau.5c00162)
Supplement: Supplementary file 1 [file mg5c00162_si_001.pdf]

Supporting Information

Boosting the mechanical strength and photocatalytic activity of 3D-printed titania aerogels by atomic layer deposition and heat treatment

Malte M. Schmidt, Tjark L. R. Gröne, Robert Zierold, Diego Ribas Gomes, Sandra König, Michael Fröba, Kaline P. Furlan, Dorota Koziej

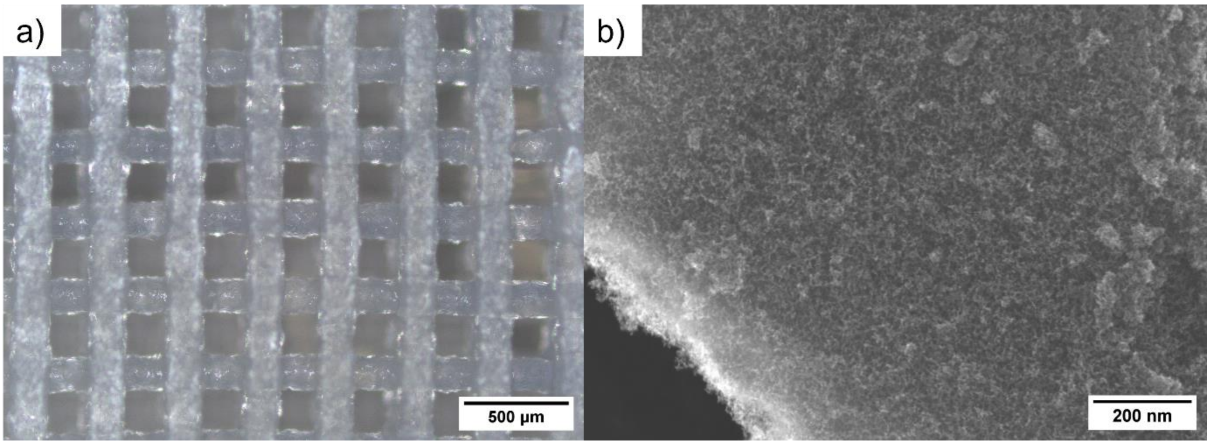

Figure S1. (a) Light microscopic image of an uncoated TiO_2 aerogel 3D-printed in a simple grid structure. (b) SEM image of an uncoated TiO_2 aerogel.

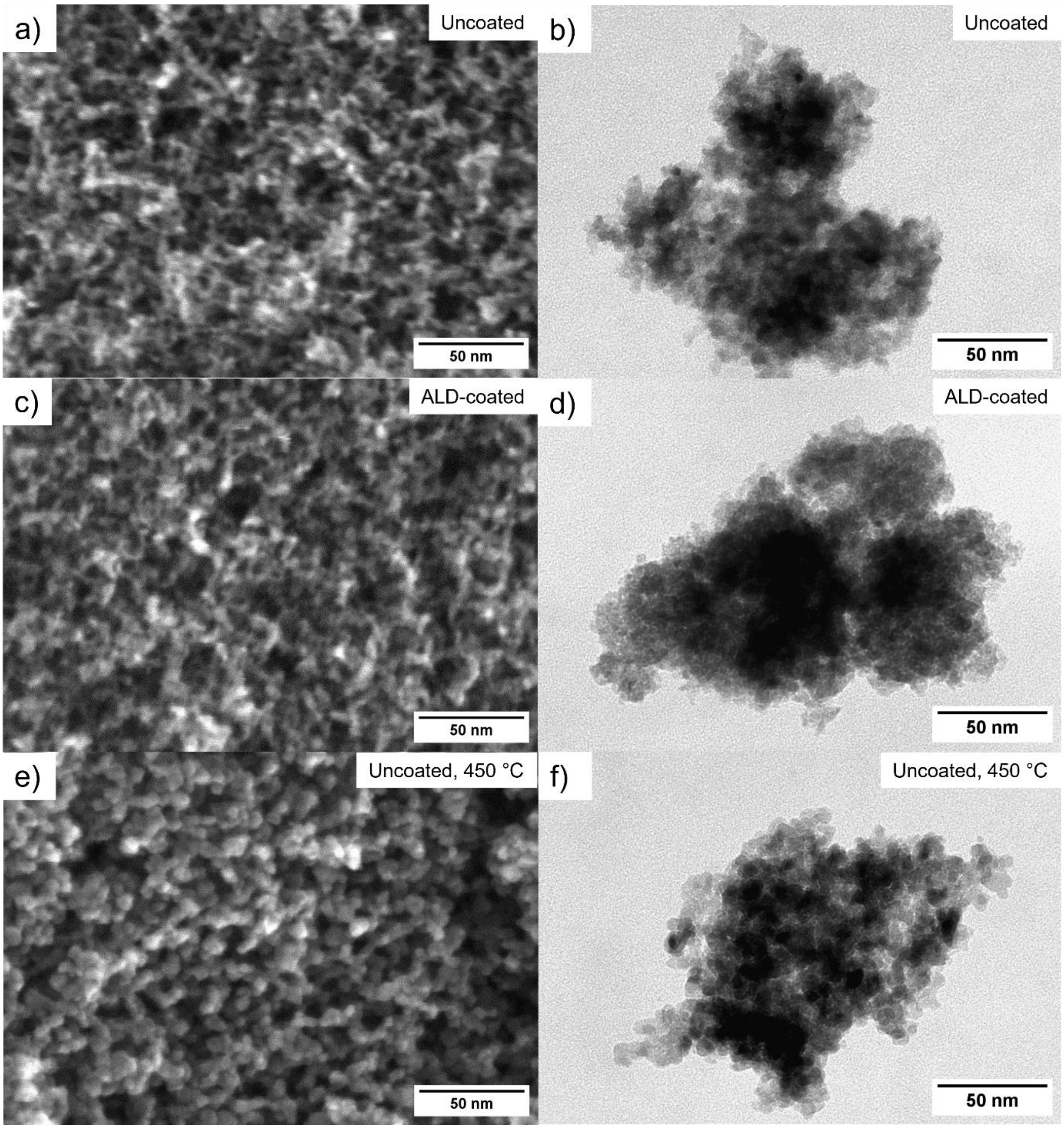

Figure S2. SEM (left) and TEM (right) images of an uncoated TiO_2 aerogel (a,b), an ALD-coated TiO_2 aerogel (c,d), and an uncoated calcined TiO_2 aerogel at 450 °C (e,f).

Table S1. Specific surface area determined from BET analysis and mean pore size determined from DFT analysis of the desorption branch of the isotherms in **Figure 2**a for an uncoated, an ALD-coated, and a calcined 3D-printed TiO_2 aerogel at 450 °C. We attribute the increase in surface area after ALD coating to variability between initial uncoated samples, whereas the decrease in surface area after calcination appears to be a systematic trend.

**TiO_2 aerogel sample**	**Specific surface area [m²/g]**	**Mean pore diameter [nm]**
Uncoated	648	22
ALD-coated	757	23
Uncoated, 450 °C	383	16
ALD-coated, 450 °C	210	18

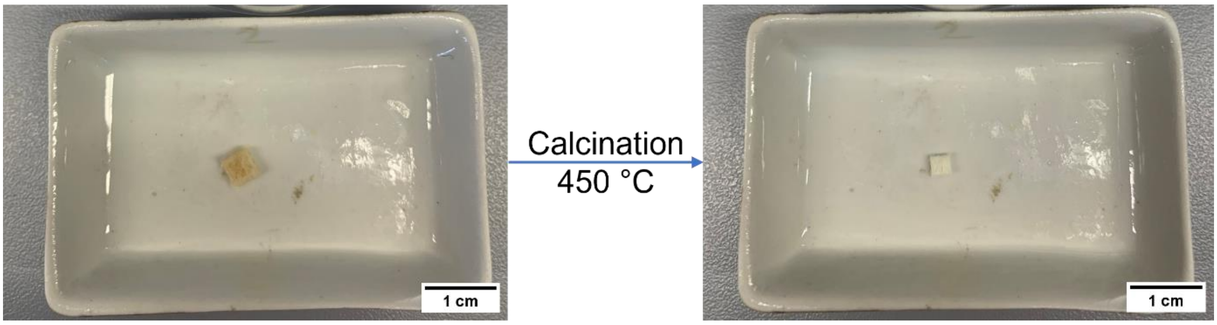

Figure S3. Photograph showing an ALD-coated TiO_2 aerogel before and after calcination at 450 °C. The gel shrinks by about one third in terms of edge length.

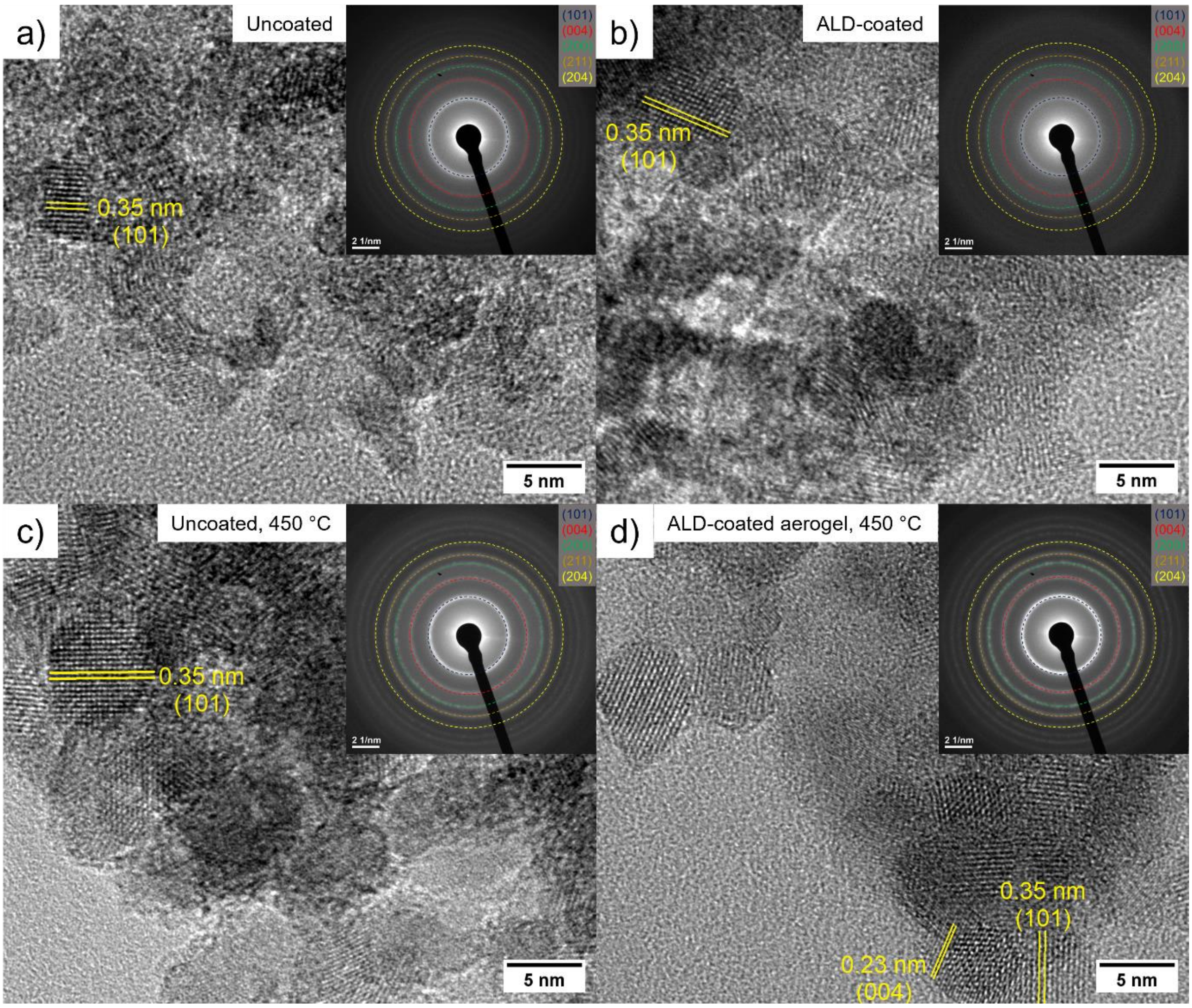

Figure S4. HR-TEM images of an uncoated (a) and an ALD-coated (b) aerogel before calcination, and an uncoated (c) and an ALD-coated (d) aerogel after calcination at 450 °C. The lattice fringes of some crystallites are highlighted, corresponding to lattice planes of anatase. The insets show electron diffraction patterns corresponding to overview images from the same sample.

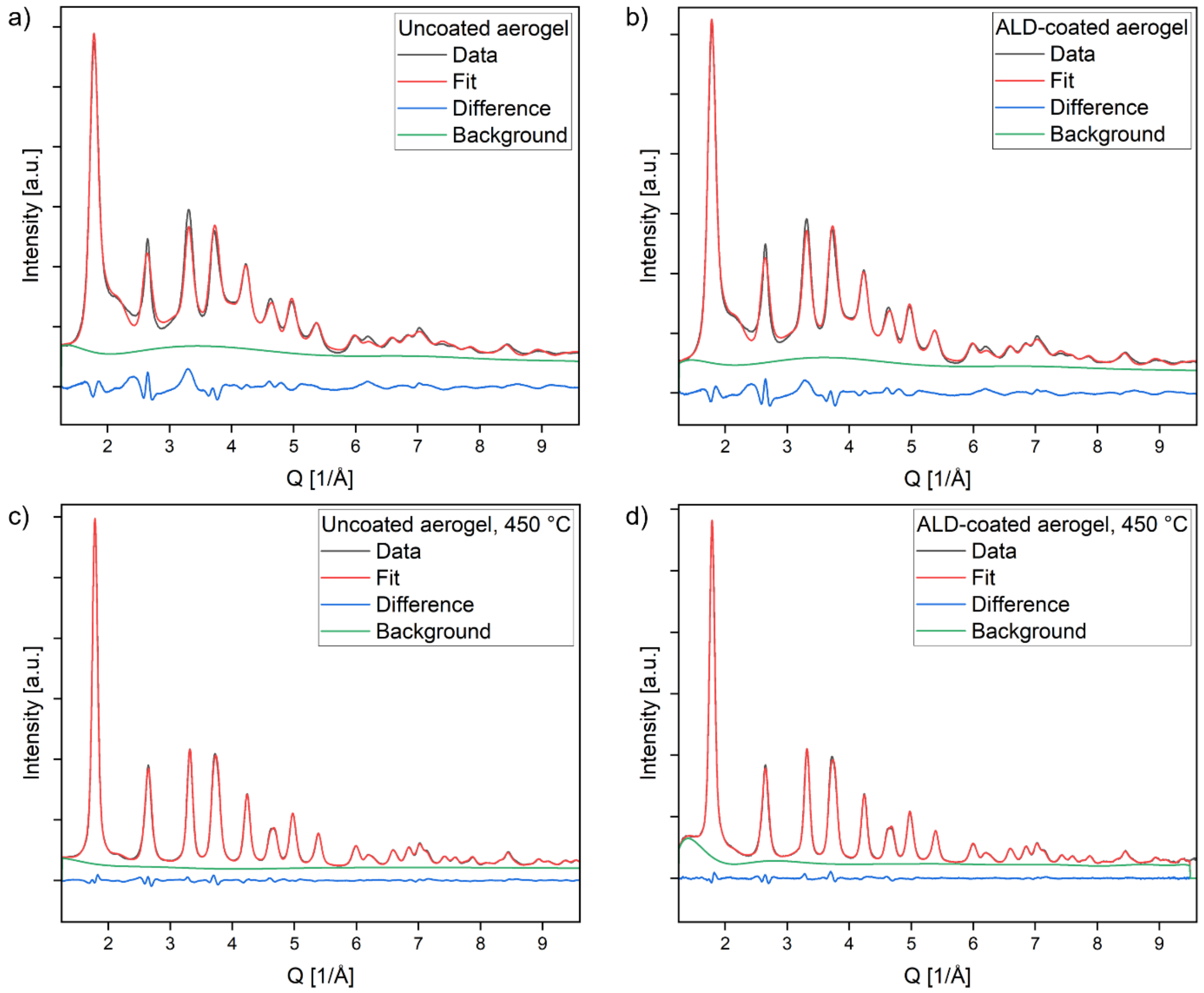

Figure S5. Background-subtracted PXRD pattern of an uncoated (a), an ALD-coated (b), an uncoated calcined (c), and an ALD-coated calcined (d) TiO_2 aerogel at 450 °C. The experimental PXRD patterns were fitted with a mixture of anatase and brookite using Rietveld refinement. The difference between experimental data and fit is plotted in blue, the background in green.

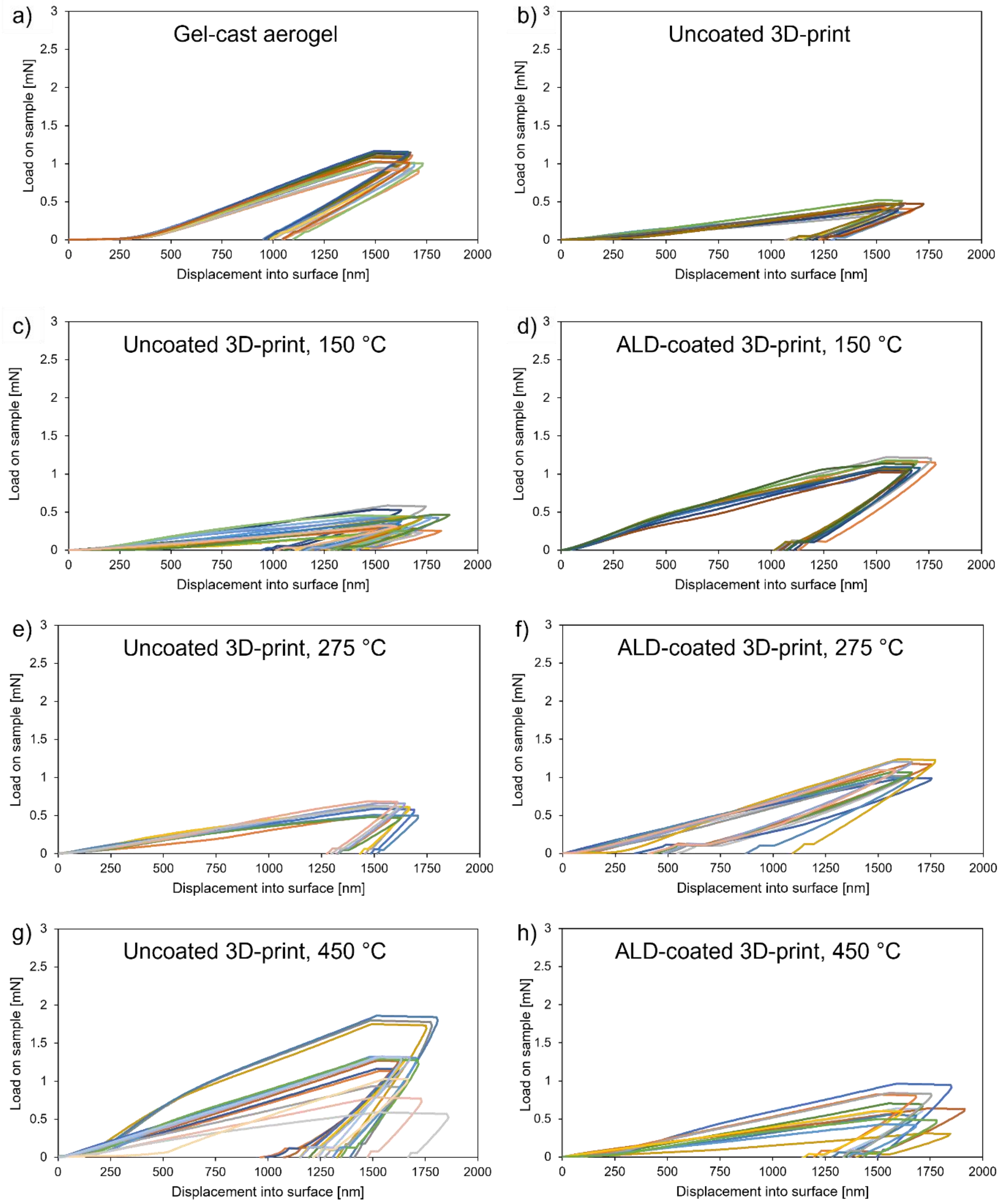

Figure S6. Load-displacement curves from nanoindentation measurements with a spherical tip in continuous stiffness mode. (a) Gel cast, monolithic aerogel, (b) uncoated 3D-printed aerogel, (c) uncoated 3D-printed aerogel heat treated under similar conditions as ALD-coating, (d) and an ALD-coated 3D-printed aerogel, (e) uncoated and (f) ALD-coated 3D-printed aerogel calcined at 275 °C, and (g) uncoated and (h) ALD-coated 3D-printed aerogel calcined at 450 °C.

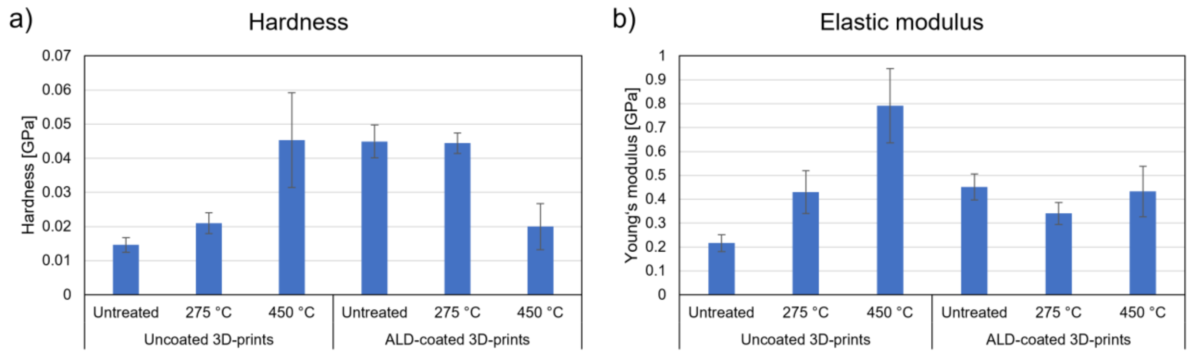

Figure S7. Hardness (a) and Young's modulus (b) comparing uncoated (left) and ALD-coated (right) samples. Each group includes untreated samples and those calcined at 275 °C or 450 °C. Error bars represent standard deviations.

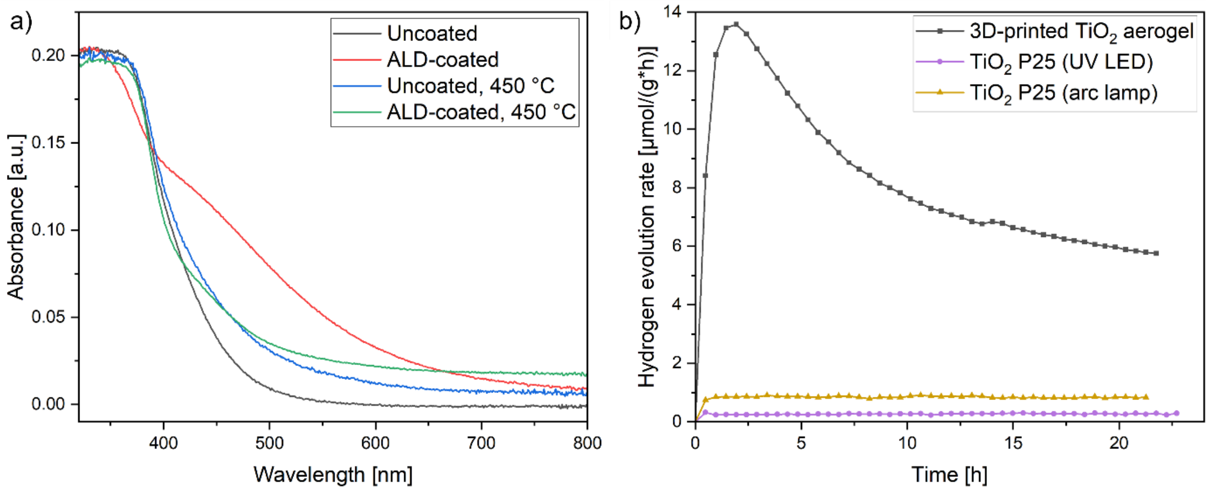

Figure S8. (a) UV/VIS absorbance spectrum of an uncoated (grey), an ALD-coated (red), uncoated calcined (blue) and ALD-coated calcined (green) TiO_2 aerogel, measured in diffuse reflectance mode. (b) Comparison of photocatalytic hydrogen production over 24 hours between an uncoated, 3D-printed TiO_2 aerogel and commercial TiO_2 P25 powder. The aerogel under 375 nm UV LED illumination (grey squares) outperforms TiO_2 P25 both under both UV LED (purple circles) and Hg/Xe arc lamp (gold triangles).
